# Do Photopletysmographic Parameters of Arterial Stiffness Differ Depending on the Presence of Arterial Hypertension and/or Atherosclerosis?

**DOI:** 10.3390/s24144572

**Published:** 2024-07-15

**Authors:** Izabela Szołtysek-Bołdys, Wioleta Zielińska-Danch, Danuta Łoboda, Krzysztof S. Gołba, Beata Sarecka-Hujar

**Affiliations:** 1 Department of General and Inorganic Chemistry, Faculty of Pharmaceutical Sciences in Sosnowiec, Medical University of Silesia in Katowice, 41-200 Sosnowiec, Poland; iboldys@sum.edu.pl (I.S.-B.); wzdanch@sum.edu.pl (W.Z.-D.); 2 Department of Electrocardiology and Heart Failure, Medical University of Silesia in Katowice, 40-635 Katowice, Poland; dana.loboda@gmail.com (D.Ł.); kgolba@sum.edu.pl (K.S.G.); 3 Department of Electrocardiology, Upper-Silesian Medical Centre in Katowice, 40-635 Katowice, Poland; 4 Department of Basic Biomedical Science, Faculty of Pharmaceutical Sciences in Sosnowiec, Medical University of Silesia in Katowice, 41-200 Sosnowiec, Poland

**Keywords:** arterial stiffness, atherosclerosis, arterial hypertension, COVID-19, SARS-CoV-2 infection

## Abstract

Background: Hypertension and atherosclerotic cardiovascular diseases (ASCVD) increase cardiovascular risk and worsen patients’ prognoses. One early predictor of increased risk is a change in arterial stiffness. This study aimed to evaluate arterial stiffness parameters using the non-invasive photoplethysmography (PPG) method in Polish patients with arterial hypertension (AH) and/or atherosclerosis (AS). Methods: The study group consisted of 333 patients (Caucasians, both sexes, aged 30–85 years old). Patients were analyzed in four groups depending on AH and AS (Group I: patients without AH or AS, Group II: AH patients, Group III: AS patients, and Group IV: AH/AS patients) and, in addition, according to sex and history of SARS-CoV-2 infection. Arterial stiffness parameters, i.e., reflection index (RI), peak-to-peak time (PPT), and stiffness index (SI) were automatically calculated with PPG based on the analysis of the pulse wave contour. Results: Mean values of RI and SI were higher in men than women (*p* < 0.001 each). Diastolic blood pressure (DBP) also differed between sexes (*p* = 0.010). Mean SI values differed between the study groups (*p* = 0.038) with the highest SI found in AS/AH patients and the lowest—in patients without AH or AS. The mean SI values were significantly lower in women compared to men in both Group I and Group II (*p* = 0.006 and *p* < 0.001, respectively). The mean values of RI were also greater in men than in women in Group I and Group II (*p* < 0.001 for each group). Regarding COVID-19 history, only HR values differed between patients with and without COVID-19 in AH patients (*p* = 0.012). In AH patients, men had higher values of RI and SI compared to women (*p* < 0.001 and *p* < 0.001). On the other hand, AS women with COVID-19 had significantly greater mean values of SI (9.66 m/s ± 1.61) than men with COVID-19 (7.98 m/s ± 1.09) (*p* = 0.045). Conclusions: The present study confirmed that sex had a significant impact on arterial stiffness parameters. Both AH and AS affected arterial stiffness. Heart rate was greater in hypertensive patients after COVID-19 compared to hypertensive patients without COVID-19.

## 1. Introduction

Today, people face constant stress and rush every day while consuming huge amounts of processed food, which affects their health, especially their cardiovascular systems. Diseases that result from this lifestyle and increase the risk of cardiovascular diseases include hypertension, diabetes, dyslipidemia, and insulin resistance. One of the symptoms of changes occurring in the arteries is a change in their stiffness. The term “arterial stiffness” is descriptive and encompasses qualitative characteristics that determine the ability of a vessel to change diameter under the influence of blood pressure. It is related to two qualities of the arteries: stiffness and elasticity. High arterial stiffness can increase systolic and diastolic blood pressure, which may be associated with increased morbidity and mortality from coronary artery disease. Reduced elasticity of arterial walls increases pulse pressure and causes damage to blood vessels, leading to increased cardiac output, which may cause left ventricular hypertrophy and diastolic dysfunction.

Increased arterial stiffness is a physiological part of the aging process. It is also strongly associated with hypertension, which is the main risk factor for developing coronary artery disease (CAD), stroke, and heart failure [1]. Effectively reducing blood pressure helps reduce this risk. In addition to aging and hypertension, one of the main causes of arterial stiffening is inflammation related to atherosclerosis (AS), arterial calcification, and oxidative stress [2,3,4,5]. AS results from dysfunction of the vascular endothelium and is a chronic process [6]. Atherosclerotic cardiovascular disease (ASCVD) is caused by plaque buildup in the arterial walls and refers to conditions that include (1) CAD, such as myocardial infarction, angina, and coronary artery stenosis; (2) cerebrovascular disease, such as a transient ischemic attack, ischemic stroke, and carotid artery stenosis; (3) peripheral artery disease, such as claudication; and (4) aortic atherosclerotic disease, such as abdominal aortic aneurysm and descending thoracic aneurysm. The increase in the incidence of cardiovascular diseases may also result from the SARS-CoV-2 virus pandemic, which lasted between 2019 and 2023. SARS-CoV-2 infection causes thromboembolic complications and persistent endothelial dysfunction, starting in the acute phase of COVID-19 and lasting several weeks/months after recovery, contributing to the development of post-COVID-19 syndrome [7,8]. Therefore, COVID-19-related vasculitis may affect arterial stiffness.

Most methods that assess the condition of arteries are invasive and carry a certain risk of complications. The gold standard for non-invasive assessment of arterial stiffness is the assessment of pulse wave velocity (PWV) using magnetic resonance imaging or by measuring Doppler blood flow velocity [9,10,11,12]. However, thanks to modern technologies, new non-invasive methods are being developed and becoming available to patients [13]. Currently, the PPG method of assessing arterial stiffness is increasingly used in early screening for CAD risk. This optical technology measures changes in the blood volume in tissues’ microvascular beds by estimating changes in the amount of light absorbed by the tissue [10]. The method has many advantages: it is simple, inexpensive, and attractive due to the device’s portability, and arterial stiffness can be assessed independently of the operator. The measurement of arterial stiffness has a prognostic value in the incidence of cardiovascular events, even in the absence of clinically apparent disease.

This study aimed to evaluate arterial stiffness parameters using the PPG method in Polish patients with AH and/or AS. We also analyzed the sex and COVID-19 history-adjusted mean values of arterial stiffness parameters in each study group.

## 2. Materials and Methods

### 2.1. Study Group

This study was of cross-sectional design and was conducted from 2021 to 2023. Analyses were performed on 333 patients (Caucasians, both sexes, 30–85 years old) who were recruited from two medical centers in Poland, the Cardiac Rehabilitation Department of the Ustroń Health Resort in 2021–2022, and the Department of Electrocardiology, Upper-Silesian Medical Center in Katowice in 2022–2023.

This study included consecutive patients who agreed to participate and consented to a non-invasive assessment of arterial stiffness. Pregnant or breastfeeding women were excluded. Also, active COVID-19 disease was grounds for exclusion from this study. Originally, 357 patients agreed to participate in this study, but for 24 patients, we were unable to obtain arterial stiffness measurement results and, therefore, they were not included in the overall analysis.

The study protocol complied with the ethical guidelines of the 1975 Declaration of Helsinki. It was approved by the Bioethical Committee of the Medical University of Silesia in Katowice, Poland (Approval No. PCN/CBN/0052/KB1/68/I/21/22, issued on 15 May 2021).

### 2.2. Division of the Study Group into Subgroups

**Figure 1:**
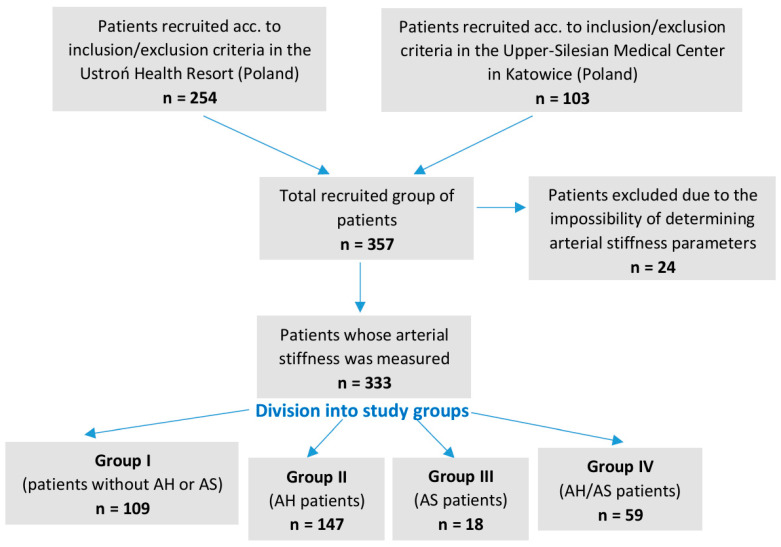
Study design with different patient groups.

Patients were analyzed in four groups depending on the presence of AH and/or AS: Group I—Reference Group (patients without AS and AH), Group II—AH patients, Group III—AS patients, and Group IV—AH/AS patients (Figure 1). AH was defined as systolic blood pressure (SBP) ≥ 140 mmHg or diastolic blood pressure (DBP) ≥ 90 mmHg or the usage of antihypertensive drugs. AS was found in patients when CAD was diagnosed, with or without myocardial infarction, or ischemic stroke.

In addition, study groups were divided into subgroups according to the following:

(1) sex (men/women);
(2) history of SARS-CoV-2 infection (yes/no).

### 2.3. Measurement of Laboratory Parameters

Routine laboratory tests were performed for total cholesterol (TC), low-density lipoprotein (LDL) cholesterol, high-density lipoprotein (HDL) cholesterol, triglycerides (TG), and D-dimer concentrations. Analyses were performed using commercially available kits.

### 2.4. Blood Pressure Measurements

Arterial blood pressure was measured using a digital electronic tensiometer model Intellisense M3 Omron (OMRON Healthcare, Milton Keynes, UK), following the 2018 ESC/ESH Clinical Practice Guidelines for the Management of Arterial Hypertension [14].

### 2.5. Measurement of Arterial Stiffness

Arterial stiffness was measured with a non-invasive PPG technique using a portable PulseTrace PCA2 meter (Micro Medical Ltd., Rochester, Kent, UK). This mains/battery-operated device allows for analysis of the pulse wave contour based on changes in blood volume in the fingertip. PPG sensors include a photo emitter (PE) and a photodetector (PD). The method involves extracting optical properties from the skin’s microvascular bed and modulating pulsatile blood flow with infrared light of the wavelength range 600–1200 nm. Light falling on the skin can be absorbed, scattered, and reflected by human tissues. The meter ensures quick (pulse waveform is obtained during a 10–15 s measurement) and operator-independent testing and is recommended by the European Network for Non-Invasive Investigation of Large Arteries for the local measurement of arterial stiffness [15].

**Figure 2:**
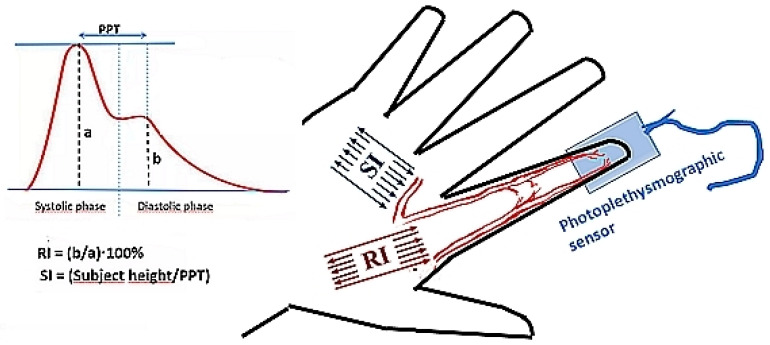
Diagram illustrating the method of determining arterial stiffness parameters: reflectance index (RI) and stiffness index (SI). During measurement, the time delay between the systolic peak (*a*) and diastolic peak (*b*) is assessed as peak-to-peak time (PPT, diagram on the left), and heart rate (HR) is measured as well.

When the heart contracts, blood is ejected from the left ventricle. A pulse is created and propagates (spreads) along the arteries. As the pulse spreads toward the lower parts of the body, the pulse wave is reflected in places where the diameter of the vessels changes and they bifurcate. The reflected waves are added, and a uniform reflected wave is created, returning to the aorta. From there, it spreads toward the finger. A typical digital volume pulse (DVP) obtained by the PulseTrace PCA2 meter is shown in Figure 2. The shape of the DVP depends on the tension and stiffness of the arteries. The first peak results from systolic blood flow and a pressure wave that starts from the left ventricle and ends at the finger. In turn, the second peak is mainly due to reflected pressure waves coming from the lower parts of the body [16]. The time of appearance of the reflected wave in relation to the direct wave depends on the stiffness of the large artery [17].

Based on the analysis of the pulse wave contour, three parameters are automatically calculated, i.e., RI as a percentage of the maximum height of the diastolic to the systolic component; PPT, which is the time between the maximum peak values of the systolic and diastolic components; and SI, which is calculated from the ratio of the patient’s height to PPT. Since the stiffness of large arteries determines the speed of the pulse wave, this parameter influences how quickly the diastolic component follows the systolic component. In addition, the HR is also measured during the assessment of arterial stiffness.

The preparation for the PPG measurement was performed according to the manufacturer’s recommendation. Before the measurement, the patient rested in a semi-supine position for approximately 15 min in a quiet, darkened room to minimize measurement interference. The temperature in the room ranged from 22–25 °C. During PPG measurement, all patients (patient and hand should be warm) lay semi-supine on a medical couch or in a hospital bed with their arms along their torsos and legs uncrossed. This position ensured a constant distance of the index finger from the heart during the examination. We made five independent measurements of arterial stiffness for each patient with intervals of app. 30 s, and the average value was calculated from these measurements.

### 2.6. Statistical Analyses

Statistical analyses were performed using Statistica 13 (STATSOFT; Statistica, Tulsa, OK, USA). The normality of the variable distribution was assessed using the Shapiro–Wilk W test. The continuous variables were expressed as mean (M) and standard deviation (SD), while categorical variables were shown as absolute numbers (*n*) and percentages (%). The comparisons of continuous variables between the two groups were made using the Student’s *t*-test or the Mann–Whitney U test, depending on the normality distribution or its lack, respectively. ANOVA or the Kruskal–Wallis H tests were used to compare continuous variables between more than two patient groups, depending on the variable’s distribution. The stochastic independence χ^2^ test was used to compare categorical variables between the study groups. Pearson’s correlation coefficients, r, were computed to assess the relationships between arterial stiffness parameters and selected variables.

In the analysis, we used also both univariate and multivariate regression models. In univariate regression models, we correlated RI and SI parameters with one out of the following 14 predictors: age, body mass index (BMI), carbon oxide (CO) in exhaled air, SBP, DBP, HR, gender, smoking status, occurrence of AS, AH, diabetes, dyslipidemia, and COVID-19. We estimated two multivariate regression models, the first for the SI parameter and the second for the RI parameter. In multivariate regression models, we used predictors significantly correlated with arterial stiffness parameters in univariate regression. We used a forward step-by-step method to select covariates into regression models. Results were regarded as significant when *p* < 0.05.

## 3. Results

### 3.1. Characteristics of the Study Group

Table 1 shows general characteristics with mean values of biochemical parameters in the total study group and sex subgroups. We observed that TC, LDL, and HDL levels were significantly higher in women than men. In turn, TG levels were slightly higher in men, and the difference was close to the significance bound. Mean values of arterial stiffness parameters, i.e., RI and SI, were higher in men than women. DBP also differed between sexes.

In the case of the accompanying diseases, the percentage of men with diabetes mellitus was almost two-fold higher than the percentage of women (*p* = 0.012). On the contrary, heart failure occurred almost four-fold higher in women than in men (*p* < 0.001) (Table 2).

Table 3 presents general characteristics and arterial stiffness parameters in the analyzed groups of patients established in dependence on the presence of AH and AS. Age, BMI, and smoking status significantly differentiated the analyzed groups. The mean values of the SI parameter were the highest in Group IV of AH/AS patients, while the lowest were in Group I. As expected, the SBP and DBP were the highest in the Patients AH. The lowest SBP was observed in Group I, while the lowest DBP was observed in Group IV (AH/AS).

The prevalence of all comorbidities for the four study groups is shown in Table 4. Diabetes mellitus was the most common in patients from Group IV (AH/AS). The lowest percentage of patients with a history of COVID-19 was found in Group IV (AH/AS). The frequency of asthma and/or chronic lung disease did not differ between the groups. Patients suffering from heart failure were the most prevalent in AH/AS patients and AS patients.

### 3.2. Sex-Adjusted Analysis of Stiffness Parameters

**Figure 3:**
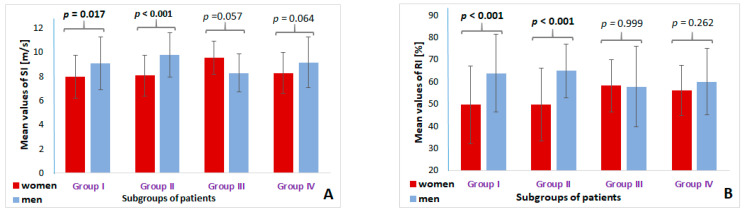

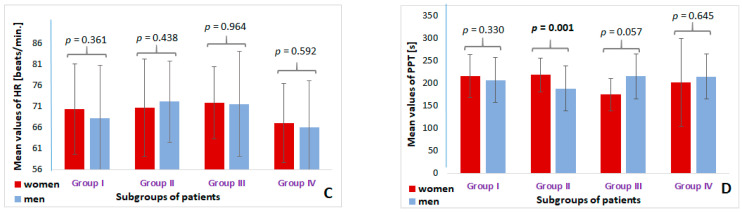
Diagrams showing sex-adjusted differences in arterial stiffness parameters ((**A**) shows SI parameter; (**B**) shows RI parameter; (**C**) shows HR parameter; (**D**) shows PPT parameter) in each distinguished study group. Group I—patients without AH or AS; Group II—AH patients; Group III—AS patients; Group IV—AH/AS patients.

The sex-adjusted mean values of arterial stiffness parameters were analyzed in each distinguished study group. Figure 3 shows diagrams of the mean SI, RI, HR, and PPT values in the study groups. The mean SI values were significantly lower in women compared to men in Group I (patients without AH or AS) and Group II (AH patients). In AH/AS patients (Group IV), the difference in SI value between sexes was close to the bound of significance (*p* = 0.064). Similarly, AS women had higher SI values compared to men, but still, the difference was close to significance (*p* = 0.057) (Figure 3A). Similar trends were observed in the case of the RI parameter’s mean values, which were significantly higher in men than in women in Group I and Group II (*p* < 0.001 for each group), while in Group III (AS patients), women and men had comparable RI values (Figure 3B). The PPT parameter was significantly greater in AH women than in men AH (*p* = 0.001), while the differences in the remaining groups were not significant (Figure 3D). The HR did not differentiate women from men in any of the study groups of patients (Figure 3C).

### 3.3. COVID-19 History-Adjusted Analysis of Stiffness Parameters

**Figure 4:**
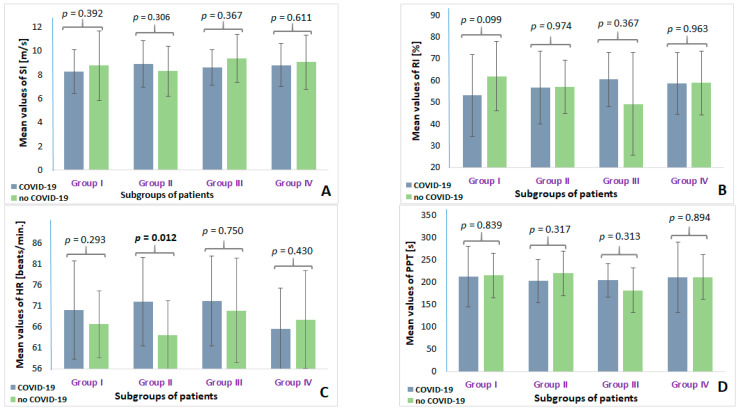
Diagrams showing COVID-19 history-adjusted differences in arterial stiffness parameters ((**A**) shows SI parameter; (**B**) shows RI parameter; (**C**) shows HR parameter; (**D**) shows PPT parameter) in each distinguished study group. Group I—patients without AH or AS; Group II—AH patients; Group III—AS patients; Group IV—AH/AS patients.

When COVID-19 history-adjusted analysis of arterial stiffness parameters was performed in each of the distinguished study groups, the only statistical difference in HR values between patients with COVID-19 and those without COVID-19 was observed in Group II of AH patients. Figure 4 shows diagrams of mean values of stiffness parameters in the COVID-19 history-adjusted subgroups from each analyzed study group. The AH patients with COVID-19 had significantly higher mean HR compared to AH patients without histories of COVID-19 (72.02 ± 10.63 beats/min vs. 64.03 ± 8.17 beats/min, *p* = 0.012) (Figure 3C). The remaining arterial stiffness parameters, i.e., SI, RI, and PPT, did not differ between COVID-19 history subgroups in other distinguished groups of patients.

In patients with COVID-19 from Group I, both mean RI and SI values were significantly higher in men than women (*p* = 0.001 and *p* = 0.045). Similar results were demonstrated in Group II (AH patients), with higher values of RI and SI in men than in women (*p* < 0.001 and *p* < 0.001). On the other hand, women with COVID-19 from Group III (AS patients) had significantly greater mean values of SI (9.66 m/s ± 1.61) than men with COVID-19 (7.98 m/s ± 1.09) (*p* = 0.045). SI values also differentiated men from women among no-COVID-19 patients from Group II, values being higher in men than in women (10.36 m/s ± 1.68 vs. 7.26 m/s ± 1.41, respectively; *p* = 0.014). In Group I, women without COVID-19 had higher HR than men without COVID-19 (71.40 beats/min ± 5.53 vs. 60.30 beats/min ± 6.28, *p* = 0.0012).

No differences in mean values of arterial stiffness parameters between men and women with or without COVID-19 were observed in Group IV.

### 3.4. Correlations between Arterial Stiffness Parameters and Blood Pressure Parameters

Table 5 shows the correlation coefficients between arterial stiffness parameters (SI, RI, PPT) and blood pressure parameters (HR, SBP, DBP) in women and men. In women, negative correlations were observed between RI and HR (r = −0.434) and PPT and HR (r = −0.167). In men correlations similar to those observed in women were revealed. In addition, DBP correlated positively with SI (r = 0.295) and RI (r = 204) and negatively with PPT (r = −0.264).

In Table 6, correlation coefficients between arterial stiffness parameters and HR, SBP, and DBP in the distinguished groups of patients are shown. In Group 1 (without AS and AH) we observed only a negative correlation between RI and HR (r = −0.496). In Group II, each arterial stiffness parameter correlated with another blood pressure parameter in a different way, i.e., SI showed a positive correlation with SBP (r = 0.170), and RI and PPT correlated negatively with HR (r = −363 and r = −178, respectively). In patients AS (Group III), RI correlated negatively with HR (r = −0.486). In turn, patients AS/AH showed the highest positive correlations between SI and HR and DBP (r = 0.482 and r = 0.503). Also, RI correlated positively with DBP (r = 0.268). In turn, PPT correlated negatively with HR and DBP (r = −0.488 and r = −0.375) while positively with SBP (r = 0.304).

### 3.5. Univariate and Multivariate Regression Models

We considered two parameters of arterial stiffness, i.e., RI and SI, with the following 14 predictors: age, BMI, COex, SBP, DBP, HR, gender, smoking status, occurrence of AH, AS, diabetes, dyslipidemia, and COVID-19. An analysis of correlation was performed between each arterial stiffness parameter with each estimated predictor. The RI parameter correlated only with two predictors, i.e., gender (*p* < 0.001) and DBP (*p* = 0.018), while the SI parameter correlated with six predictors: age (*p* < 0.001), CO (*p* = 0.049), DBP (*p*< 0.001), gender (*p* < 0.001), smoking status (*p* = 0.035), and hypertension (*p* = 0.031). A multivariate regression for RI revealed that only gender correlated significantly with this parameter (b = 12.104, *p* < 0.001). This means that men had a higher RI parameter by 12.014% on average. In the case of the SI parameter, multivariate regression revealed three significant predictors, namely age (b = 0.039, *p* < 0.001), DP (b = 0.047, *p* < 0.001), and gender (b = 1.039, *p* < 0.001). An increase in age by one year causes an increase in the value of the SI parameter by 0.039 m/s on average, an increase in DBP by 1 mmHg causes an increase in SI by 0.047 m/s on average, and men have higher SI by 1.039 m/s on average than women.

## 4. Discussion

In the present study, we assessed the values of arterial stiffness parameters (i.e., RI, SI, and PPT) in patients with cardiac diseases using photoplethysmography. In the total study group, men had significantly higher RI and SI values and lower PPT values than women. When we divided patients into four groups according to the presence of hypertension and/or atherosclerosis, SI was the only parameter that differed between groups. The mean RI and PPT values were comparable between all four groups. The mean SI and RI values were significantly lower in women compared to men among both patients without AH or AS and AH patients. When COVID-19 history-adjusted analysis of arterial stiffness parameters was taken into account, the only statistical difference between patients with COVID-19 and those without COVID-19 was observed in the case of mean HR values among patients AH. In addition, we observed correlations both positive and negative of arterial stiffness parameters with HR, SBP, and DBP in sex subgroups and groups of patients distinguished on the presence of AH and/or AS. In our study, multivariate regression analysis revealed that only gender correlated significantly with RI, while age, DBP, and gender correlated positively with SI. The association between SI and age in multivariate analysis was previously reported in several studies [18,19,20]. In the study by Gunarathne et al. [18], multivariate regression analysis showed that age was independently correlated with SI. In turn, Iranian research by Alaei-Shahmiri et al. [19] also showed a relationship between SI and age in a multiple linear regression analysis; however, in contrast to our results, the authors observed a relationship between SI and SBP and, in our study, DBP was associated with SI parameters.

Arterial stiffness parameters measured by PPG are influenced by many factors. In addition to those already established, such as age, sex, hypertension, and atherosclerotic cardiovascular diseases (ASCVD) [20,21], arterial stiffness can depend on inflammatory diseases [22].

Millasseau et al. [21] showed that SI increases with the age of the subjects. The authors found this based on the time delay between the peaks of systole and diastole, which decreases with age due to the increased stiffness of large arteries. According to the authors, the PPT and the stiffness index (SI = h/PPT) are the best features for accurately classifying cardiovascular diseases using the first derivative of PPG [21]. In our previous analysis of Polish patients with long-COVID syndrome, we found that age was also one of the critical determinants of arterial stiffness in post-COVID convalescents [23]. In the present study, this association is also confirmed.

Sex is also an important factor influencing arterial stiffness. In the population we studied, sex was the most important determinant differentiating arterial stiffness parameters. In the whole group, RI and SI were higher for men than women. The PPT parameter shows less variation, but introducing height as a standardizing factor (SI = h/PPT) allows for statistically significant differences between the arterial stiffness of men and women from each analyzed group. However, this relationship may not apply to young adults [24] and people aged <40 years [25]. In the study based on a cohort from the UK composed of healthy volunteers and people with established CVD risk factors, the SI parameter was significantly higher in males [18]. In addition, the authors observed a significantly higher mean SI in people with CVD risk factors than in healthy people, both in men and women [18].

Another determinant of artery stiffening is arterial hypertension. In our cohort, the SI value was the highest in patients with AH compared to patients without AH or AS. The pathophysiology of both conditions, i.e., arterial stiffness and hypertension, is related. Hypertension leads to damage to the vessel walls, which may result from mechanical stress, endothelial dysfunction, or inflammation. The arteries, which then stiffen in response to changes in blood pressure, increase systolic blood pressure, which further hardens the arteries and pulse pressure [26]. Earlier, a significant relationship between SI and blood pressure parameters was demonstrated [25]. In the study by Jeya Shree et al. [27], arterial stiffness determined by the PPG method was compared between people with hypertension and healthy people. SI in healthy men (32 years ± 2) was 9.01 m/s ± 2.55, and it was 11.2 m/s ± 2.51 in men with hypertension (34 years ± 6) [27]. These values differ significantly from those we observed in our work. The men examined in our group were much older, and the SI values in people without hypertension compared to people with hypertension were 9.45 m/s ± 2.24 and 10.36 m/s ± 1.69, respectively, so these values were lower than in the above-mentioned study. In the case of women participating in our study, they were mainly in the peri- or postmenopausal period; hence, the values of arterial stiffness parameters were higher than those observed by Madhura et al. [28]. The authors reported changes in the SI and RI parameters in women who were examined according to their menstrual cycle demonstrating a significant decrease in these parameters during the mid-cycle [28].

The presence of ASCVD also determines the values of arterial stiffness parameters in both sexes. The mean value of SI was significantly higher in women with diagnosed AS than in women with AH (9.53 m/s ± 1.37 vs. 8.05 m/s ± 1.70, respectively; *p* = 0.023). The opposite trend was found in men. In men with AS, SI was lower than in men with AH (8.26 m/s ± 1.57 vs. 9.76 ± 1.85 m/s, respectively; *p* = 0.011). In this study, we also found some correlations between SI, RI, and PPT depending on the sexes. Among women, RI correlated negatively with HR (r = −0.434) and PPT with HR (r = −0.167). In men, correlations similar to those observed in women were revealed, and DBP correlated positively with SI (r = 0.295) and RI (r = 0.204) and negatively with PPT (r = −0.264). In the study performed in a large cohort of patients from the UK, Said et al. [29] demonstrated that a higher arterial stiffness index (ASI) measured by finger PPG was associated with an increased risk of overall CVD, myocardial infarction, coronary heart disease, and heart failure. ASI was also found as a predictor of mortality [29].

The COVID-19 pandemic, which was announced to end in 2023, showed that COVID-19 history may be a risk factor for CVD, but it was also suggested as a risk factor for arterial stiffness. Recent data by Nandadeva et al. [30] demonstrated a higher central arterial stiffness in women with post-acute sequelae of COVID-19 than in healthy controls. In the present study, the only parameter that differentiated patients with and without COVID-19 history in Group II of patients with AH was HR. Recent data on a large cohort of over 45,000 patients with COVID-19 without AH demonstrated that newly developed persistent AH is frequent in both hospitalized and non-hospitalized patients with COVID-19 (20.6% and 10.85%, respectively) [31]. In our study, a more detailed analysis of the RI value considering gender also showed differences in this parameter between men and women after COVID-19 (i.e., 62.76 % ± 18.55 and 48.76 % ± 17.45, respectively; *p* = 0.003). In patients who did not have COVID-19, the RI value was higher in the men compared, but the difference of 12% turned out to be statistically insignificant.

The literature reports that an increased resting heart rate is a risk factor in patients with coronary artery disease [32,33]. A meta-analysis of 45 nonrandomized prospective cohort studies performed by Zhang et al. [32] showed that resting heart rate was an independent predictor of CAD, stroke, and sudden death. Elevated HR and blood pressure are associated with an increased risk of cardiovascular disease in people with hypertension [34]. In the study by Brillante et al. [25], a negative correlation between RI and HR was presented, while SI correlated positively with HR. In our study, negative correlations between RI and HR were demonstrated in Group I (no AH and/or AS), Group II (only AH), and Group III (only AS), while in Group IV (AH/AS), SI correlated positively with HR.

This study has some limitations. Arterial stiffness parameters measured by PPG are influenced by the quality of the signal depending on the properties of the subject’s skin at measurement, including individual skin structure, blood oxygen saturation, blood flow rate, skin temperatures, and the measuring environment [35]. In our study, attempts were made to minimize factors such as dry skin, cold hands, noise, and light that would cause artifacts. Despite all these steps taken, we were not able to determine arterial stiffness parameters for as many as 24 people due to technical reasons.

The literature still provides scarce information regarding the SI and RI parameter values, which forced us to compare the obtained data with the values of younger, healthy people described earlier [24,25].

## 5. Conclusions

The present study confirmed that gender had a significant impact on arterial stiffness parameters. Both AH and AS affected arterial stiffness. In hypertensive patients after COVID-19, HR was increased compared to hypertensive patients without COVID-19.

## Figures and Tables

**Table 1:** General and biochemical characteristics of the total study group and the sex subgroups.

	Total Group N = 333	Females N = 176	Males N = 157	*p*
Age [years], M ± SD	60.42 ± 11.76	59.41 ± 11.50	61.55 ± 11.97	0.097
BMI [kg/m^2^], M ± SD	28.80 ± 4.99	28.54 ± 5.65	29.08 ± 4.15	0.330
Smoking status, *n* (%)				<0.001
Nonsmokers	175 (52.55)	112 (63.64)	63 (40.13)	
Smokers	28 (8.41)	12 (6.82)	18 (11.46)	
Former smokers	130 (39.04)	54 (30.68)	76 (48.41)	
TC [mg/dL], M ± SD	215.06 ± 67.07	225.93 ± 63.65	202.30 ± 68.93	0.002
LDL [mg/dL], M ± SD	127.81 ± 48.28	135.83 ± 46.27	118.57 ± 49.05	0.002
HDL [mg/dL], M ± SD	63.93 ± 25.92	70.77 ± 25.55	56.02 ± 24.12	<0.001
TG [mg/dL], M ± SD	168.00 ± 109.73	157.23 ± 80.92	180.31 ± 134.65	0.070
D-dimers [mg/L], M ± SD	0.70 ± 0.73	0.75 ± 0.73	0.63 ± 0.73	0.280
HR [beats/min], M ± SD	69.92 ± 11.00	70.24 ± 10.89	69.55 ± 11.15	0.569
SBP [mmHg], M ± SD	130.39 ± 17.25	129.46 ± 17.34	131.42 ± 17.15	0.310
DBP [mmHg], M ± SD	77.58 ± 9.01	76.36 ± 8.94	78.96 ± 8.93	0.010
SI [m/s], M ± SD	8.68 ± 1.97	8.10 ± 1.74	9.34 ± 2.00	<0.001
RI [%], M ± SD	56.34 ± 16.80	50.57 ± 16.43	62.81 ± 14.77	<0.001
PPT [s], M ± SD	207.84 ± 59.38	213.95 ± 56.38	201.00 ± 62.04	0.047

M—mean; SD—standard deviation; BMI—body mass index; TC—total cholesterol; LDL—low-density lipoprotein; HDL—high-density lipoprotein; TG—triglycerides; SBP—systolic blood pressure; DBP—diastolic blood pressure; SI—stiffness index; RI—reflection index; PPT—peak-to-peak time; HR—heart rate; M ± SD—mean ± standard deviation. Significant differences are in bold.

**Table 2:** The frequency of comorbidities in the total study group and the sex subgroups.

	Total Group N = 333	Females N = 176	Males N = 157	*p*
Diabetes mellitus, *n* (%)	78 (23.42)	31 (17.61)	47 (29.94)	0.012
Heart failure, *n* (%)	43 (12.91)	34 (19.32)	9 (5.73)	<0.001
Asthma/Chronic lung disease, *n* (%)	34 (10.21)	16 (9.09)	18 (11.46)	0.865
Chronic renal failure, *n* (%)	28 (8.41)	15 (8.52)	13 (8.28)	0.583
COVID-19, *n* (%)	276 (82.88)	152 (86.36)	124 (78.98)	0.128
Gout, *n* (%)	33 (9.91)	15 (8.52)	18 (11.46)	0.952

COVID-19—coronavirus 2019 disease; CAD—coronary artery disease.

**Table 3:** General characteristics and mean values of arterial stiffness parameters in subgroups of patients depending on the presence of AH and AS.

	Group I Patients without AH or AS N = 109	Group II AH Patients N = 147	Group III AS Patients N = 18	Group IV AH/AS Patients N = 59	*p*
Age [years], M ± SD	54.39 ± 13.00	61.39 ± 9.28	62.39 ± 10.09	68.54 ± 9.53	<0.001
BMI [kg/m^2^], M ± SD	26.67 ± 4.59	29.89 ± 4.73	28.35 ± 5.61	30.19 ± 4.89	<0.001
Smoking status, *n* (%)					0.002
Nonsmokers	64 (58.72)	85 (57.82)	4 (22.22)	22 (37.29)	
Smokers	10 (9.17)	9 (6.12)	5 (27.78)	4 (6.78)	
Former smokers	35 (32.11)	53 (36.05)	9 (50.00)	33 (55.93)	
HR [beats/min], M ± SD	69.64 ± 11.36	71.36 ± 10.66	71.68 ± 10.82	66.29 ± 10.61	0.038
SBP [mmHg], M ± SD	121.91 ± 17.48	137.35 ± 15.38	128.35 ± 15.63	129.13 ± 14.22	<0.001
DBP [mmHg], M ± SD	75.15 ± 9.17	80.53 ± 8.28	76.53 ± 9.91	74.87 ± 8.12	<0.001
SI [m/s], M ± SD	8.33 ± 1.98	8.85 ± 1.96	8.76 ± 1.59	8.90 ± 2.00	0.038
RI [%], M ± SD	54.17 ± 18.67	56.75 ± 16.43	57.88 ± 15.53	58.87 ± 14.14	0.499
PPT [s], M ± SD	212.83 ± 47.41	203.89 ± 57.04	199.30 ± 40.83	211.10 ± 84.85	0.157

AS—atherosclerosis; AH—arterial hypertension; M—mean; SD—standard deviation; BMI—body mass index; SBP—systolic blood pressure; DBP—diastolic blood pressure; SI—stiffness index; RI—reflection index; PPT—peak-to-peak time; HR—heart rate. Significant differences are in bold.

**Table 4:** The frequency of comorbidities in subgroups of patients depending on the presence of AH and AS.

	Group I Patients without AH or AS N = 109	Group II AH Patients N = 147	Group III AS Patients N = 18	Group IV AS/AH Patients N = 59	*p*
Diabetes mellitus, *n* (%)	10 (9.17)	38 (25.85)	5 (27.78)	25 (42.37)	<0.001
Heart failure, *n* (%)	5 (4.59)	10 (6.80)	5 (27.78)	23 (38.98)	<0.001
Asthma/Chronic lung disease, *n* (%)	10 (10.09)	14 (9.52)	2 (11.11)	7 (11.86)	0.967
Chronic renal failure, *n* (%)	1 (0.92)	10 (6.80)	2 (11.11)	15 (25.86)	<0.001
COVID-19, *n* (%)	95 (87.16)	135 (91.84)	14 (77.78)	32 (55.17)	<0.001
Gout, *n* (%)	2 (1.94)	18 (13.23)	1 (5.88)	12 (21.43)	<0.001

AS—atherosclerosis; AH—arterial hypertension; COVID-19—coronavirus 2019 disease; CAD—coronary artery disease.

**Table 5:** Correlation coefficients between arterial stiffness parameters (SI, RI, PPT) and blood pressure parameters (HR, SBP, DBP) in sex subgroups.

Arterial Stiffness Parameters	Women						Men					
	SI [m/s]		RI [%]		PPT [s]		SI [m/s]		RI [%]		PPT [s]	
	r	*p*	r	*p*	r	*p*	r	*p*	r	*p*	r	*p*
HR [beats/min]	0.144	0.062	**−0.434**	**<0.001**	**−0.167**	**0.030**	0.102	0.212	**−0.309**	**<0.001**	**−0.239**	**0.003**
SBP [mmHg]	0.055	0.482	0.030	0.699	−0.066	0.391	0.074	0.363	0.042	0.611	0.056	0.491
DBP [mmHg]	0.124	0.107	−0.013	0.869	−0.049	0.529	**0.295**	**<0.001**	**0.204**	**0.012**	**−0.264**	**0.001**

SI—stiffness index; RI—reflection index; PPT—peak-to-peak time; HR—heart rate; SBP—systolic blood pressure; DBP—diastolic blood pressure. Significant results are in bold.

**Table 6:** Correlation coefficients between arterial stiffness parameters (SI, RI, PPT) and blood pressure parameters (HR, SBP, DBP) in study groups based on the presence of AH and AS.

Arterial Stiffness Parameters	**Group I Patients without AH or AS**						**Group II AH Patients**					
	**SI [m/s]**		**RI [%]**		**PPT [s]**		**SI [m/s]**		**RI [%]**		**PPT [s]**	
	**r**	***p***	**r**	***p***	**r**	***p***	**r**	***p***	**r**	***p***	**r**	***p***
HR [beats/min]	−0.021	0.833	**−0.496**	**<0.001**	−0.022	0.822	0.099	0.240	**−0.363**	**<0.001**	**−0.178**	**0.033**
SBP [mmHg]	0.061	0.531	0.014	0.887	−0.052	0.601	**0.170**	**0.042**	0.144	0.085	−0.082	0.332
DBP [mmHg]	0.184	0.059	0.128	0.189	−0.145	0.138	0.144	0.085	0.108	0.200	−0.082	0.330
Arterial stiffness parameters	**Group III** AS patients						**Group IV** AH/AS patients					
	SI [m/s]		RI [%]		PPT [s]		SI [m/s]		RI [%]		PPT [s]	
	**r**	***p***	**r**	***p***	**r**	***p***	**r**	***p***	**r**	***p***	**r**	***p***
HR [beats/min]	−0.386	0.126	**−0.486**	**0.048**	0.293	0.254	**0.482**	**<0.001**	0.189	0.891	**−0.488**	**<0.001**
SBP [mmHg]	0.049	0.851	0.097	0.711	0.036	0.891	−0.103	0.453	−0.230	0.091	**0.304**	**0.024**
DBP [mmHg]	0.219	0.398	0.056	0.831	−0.184	0.478	**0.503**	**<0.001**	**0.268**	**0.048**	**−0.375**	**0.005**

AS—atherosclerosis; AH—arterial hypertension; SI—stiffness index; RI—reflection index; PPT—peak-to-peak time; HR—heart rate; SBP—systolic blood pressure; DBP—diastolic blood pressure. Significant results are in bold.

## Data Availability

The data presented in this study are available on request in the Department of Basic Biomedical Science, Faculty of Pharmaceutical Sciences, Medical University of Silesia in Katowice (Poland). The data are not publicly available due to privacy restrictions.
